# Mass spectral analysis of mouse serum proteome to predict tumor clearance

**DOI:** 10.1186/2051-1426-3-S2-P100

**Published:** 2015-11-04

**Authors:** Norman Woller, Heinrich Roder, Sarah Knocke, Krista Meyer, Julia Grigorieva, Senait Asmellash, Kevin Sayers, Caroline Maher

**Affiliations:** 1 Hannover Medical School, Hannover, Germany; 2 Biodesix Inc., Boulder, CO, USA

## Background

A novel transposon tumor induction model was used to induce cholangiocarcinoma tumors in C57BL/6 mice. In this system, it is possible to drive immune related activities that will lead to 1) large tumors, no clearance or response by tumor-directed CD8+ T cells, 2) large tumors, no clearance, with tumor-specific CD8+ response, or 3) CD8+ response, with tumor clearance. Analysis of proteomic data resulting from serum samples taken pre-inoculation and at study end were compared within and across the three groups of mice to determine if there are detectable differences attributable to immune functions or tumor-immune interactions that may be used to predict outcome.

## Methods

Tumors were induced using a transposon-plasmid harboring oncogenic Kras via hydrodynamic injection into the livers of 18 mice. In addition Group 2 mice received a primer for CD8+ response and Group 3 mice received activating signals for both CD8+ and CD4+ directed responses. Deep MALDI spectra were acquired from mouse sera collected from mice (6 per group, 2 time points) from 3-70KDa using a SimulTOF 100 from Virgin Instruments in positive ion mode. The equivalent of 400K shot spectra were created for individual mice and analyzed.

## Results

Mice in groups 1 and 2 grew intermediate or large tumors. In Group 3, 2 mice had no detectable tumor, 1 mouse had a small tumor, and 2 mice had sizable tumors. 30 MS features had differential behavior between groups. Some features correlated with tumor burden while others may be directly related to or correlated with immune function.

## Conclusions

Proteins in serum can be detected that measure immune related activities. The sophisticated model developed in the mouse system allows us to probe the serum proteome and may translate to a better understanding of the human immune-tumor interaction. We are actively working to confirm the protein identifications to present at the meeting. The study demonstrates that serum contains information related to the ability of the host to clear tumors and that the DM platform is prime for detecting these differences. The combination of methods can be used to identify candidate biomarkers from the genetically controlled model that can be translated and tested in human studies with the goal of patient selection for immunotherapies, measuring response, or to aid in identification of progression in immunotherapy treated patients.

